# Sequelae, persistent symptomatology and outcomes after COVID-19 hospitalization: the ANCOHVID multicentre 6-month follow-up study

**DOI:** 10.1186/s12916-021-02003-7

**Published:** 2021-05-20

**Authors:** Álvaro Romero-Duarte, Mario Rivera-Izquierdo, Inmaculada Guerrero-Fernández de Alba, Marina Pérez-Contreras, Nicolás Francisco Fernández-Martínez, Rafael Ruiz-Montero, Álvaro Serrano-Ortiz, Rocío Ortiz González-Serna, Inmaculada Salcedo-Leal, Eladio Jiménez-Mejías, Antonio Cárdenas-Cruz

**Affiliations:** 1 School of Medicine, University of Granada, Granada, Spain; 2 Service of Preventive Medicine and Public Health, Hospital Universitario Clínico San Cecilio, Granada, Spain; 3 Department of Preventive Medicine and Public Health, University of Granada, Avda. de la Investigación n°11, 18016 Granada, Spain; 4 Instituto de Investigación Biosanitaria, ibs.GRANADA, Granada, Spain; 5 Service of Preventive Medicine and Public Health, Complejo Hospitalario de Jaén, Jaén, Spain; 6 Service of Preventive Medicine and Public Health, Hospital Universitario de Puerto Real, Puerto Real, Cádiz Spain; 7 Unidad de Gestión Clínica Interniveles de Prevención, Promoción y Vigilancia de la Salud, Hospital Universitario Reina Sofía, Córdoba, Spain; 8 Instituto Maimónides de Investigación Biomédica de Córdoba (Imibic), Córdoba, Spain; 9 Chair of Teaching and Research in Family Medicine, SEMERGEN-UGR, University of Granada, Granada, Spain; 10 Intensive Care Unit, Hospital de Poniente, El Ejido, Almería, Spain

**Keywords:** COVID-19, Long COVID, Post-discharge, Sequelae, Persistent symptoms, Primary care, Follow-up

## Abstract

**Background:**

Long-term effects of COVID-19, also called Long COVID, affect more than 10% of patients. The most severe cases (i.e. those requiring hospitalization) present a higher frequency of sequelae, but detailed information on these effects is still lacking. The objective of this study is to identify and quantify the frequency and outcomes associated with the presence of sequelae or persistent symptomatology (SPS) during the 6 months after discharge for COVID-19.

**Methods:**

Retrospective observational 6-month follow-up study conducted in four hospitals of Spain. A cohort of all 969 patients who were hospitalized with PCR-confirmed SARS-CoV-2 from March 1 to April 15, 2020, was included. We collected all the SPS during the 6 months after discharge reported by patients during follow-up from primary care records. Cluster analyses were performed to validate the measures. The main outcome measures were return to the Emergency Services, hospital readmission and post-discharge death. Surviving patients’ outcomes were collected through clinical histories and primary care reports. Multiple logistic regression models were applied.

**Results:**

The 797 (82.2%) patients who survived constituted the sample followed, while the rest died from COVID-19. The mean age was 63.0 years, 53.7% of them were men and 509 (63.9%) reported some sequelae during the first 6 months after discharge. These sequelae were very diverse, but the most frequent were respiratory (42.0%), systemic (36.1%), neurological (20.8%), mental health (12.2%) and infectious (7.9%) SPS, with some differences by sex. Women presented higher frequencies of headache and mental health SPS, among others. A total of 160 (20.1%) patients returned to the Emergency Services, 35 (4.4%) required hospital readmission and 8 (1.0%) died during follow-up. The main factors independently associated with the return to Emergency Services were persistent fever, dermatological SPS, arrythmia or palpitations, thoracic pain and pneumonia.

**Conclusions:**

COVID-19 cases requiring hospitalization during the first wave of the pandemic developed a significant range of mid- to long-term SPS. A detailed list of symptoms and outcomes is provided in this multicentre study. Identification of possible factors associated with these SPS could be useful to optimize preventive follow-up strategies in primary care for the coming months of the pandemic.

**Supplementary Information:**

The online version contains supplementary material available at 10.1186/s12916-021-02003-7.

## Background

During the so-called first wave of the COVID-19 pandemic, a rapid progression of the infection was reported in China and worldwide [1, 2]. This progression led to a significant number of severe cases that required hospitalization and intensive care [3]. Immediately, major efforts were made internationally in order to identify the main symptoms to optimize diagnosis [4–6] and the main prognostic factors to improve treatment and healthcare strategies [7–10]. A wide and multifaceted range of clinical manifestations was identified, including respiratory, gastrointestinal, neurological and cardiovascular symptoms, among others [11].

After the first wave, new efforts focused on identifying the potential short-term sequelae following COVID-19 infection, especially in higher-risk cases requiring longer hospital care, the so-called post-discharge syndrome [12]. Among the most frequent, respiratory and neurological sequelae [13], cutaneous signs [14] or headache [15] have been described. The lethality and factors related to COVID-19 are being thoroughly analysed but, given the high number of hospitalized patients and the potential morbidity it could generate, further research on possible sequelae after hospitalization is still required.

However, there has not yet been sufficient time to evaluate the long-term effects of COVID-19 in these severe cases requiring hospitalization. To date, there are very few studies that provide detailed information on the wide range of sequelae and persistent symptoms (SPS) after 6 months of follow-up [16]. Some studies describe the presence of persistent symptoms after 3 months of follow-up [12, 17–20], but further evidence is still needed.

Furthermore, there is no information in the current literature on possible outcomes associated with these SPS (death after discharge, readmission to hospital or emergency care) that would be useful for improving preventive measures and primary care follow-up after discharge. Therefore, the design of high-risk patient profiles and the individualization of clinical follow-up according to risk and SPS could be useful for the correct management in primary care follow-up and discharge criteria, which are necessary in a context of high healthcare pressure.

The objective of this study was twofold. First, we aimed to identify and quantify the SPS during the 6 months after discharge for COVID-19. Second, we aimed to analyse the association of SPS with negative outcomes (return to the Emergency Services, hospital readmission and death) in the 6 months following discharge to plan differential preventive follow-up strategies.

## Methods

### Study design and setting

A 6-month retrospective longitudinal observational follow-up study was designed, according to Strengthening the Reporting of Observational Studies in Epidemiology (STROBE) guidelines (Additional file 1). The study sample consisted of the cohort of patients admitted to the selected hospitals in four cities in Andalusia, Spain (Córdoba, Granada, Jaén and Puerto Real) from March 1 to April 15, 2020. Inclusion criteria included confirmed polymerase chain reaction (PCR) to SARS-CoV-2 (confirmed cases according to the Spanish Ministry of Health). Therefore, patients with compatible clinical symptoms and imaging test (suspected cases) but negative PCR were not included. In addition, only patients who required hospitalization were considered (patients identified in the Emergency Services who did not require hospitalization were excluded). All cases were followed up for 6 months after discharge. Therefore, the follow-up of the study concluded on January 5, 2021.

### Data source and variables

Hospitalization medical records were consulted to collect data on sociodemographic, clinical, therapeutic and evolution factors of COVID-19 patients. To collect information on SPS after discharge, primary care records were consulted. Follow-up consultation records and periodic telephonic reports scheduled from primary care standardized in Andalusia were consulted. Data from hospital specialties (pneumology and infectious diseases services) were also consulted to obtain more information on SPS. The SPS collected occurred at any time after discharge and before the outcomes. Therefore, SPS registered on return to Emergency Services or readmission were not considered as source of SPS information, in an attempt to avoid potential selection bias. Similarly, when an outcome occurred more than once (e.g. return to the Emergency Services several times), only the SPS reported before the first return were considered for the analysis to avoid reverse causality. Finally, data on dependency and residential care centres were obtained by accessing the Andalusian Epidemiological Surveillance System (SVEA). Obesity and smoking were not considered for the analyses because most of the records did not include these variables. The variables included in the study were:

- *Sociodemographic variables*: sex, age, country, residence (living at home or at specific centres), dependency for activities of daily living (DADL)
- *Admission variables*: admission to the intensive care unit (ICU) or hospitalization, time of hospitalization
- *Clinical variables*: past medical history, analytical results, presence of concomitant infections, prognostic scores as CURB-65 (confusion, blood urea, respiratory rate, blood pressure and age>65) and the Sequential Organ Failure Assessment (SOFA), candidate to cardiopulmonary resuscitation (CPR), treatments during hospitalization, outcome (discharge or intra-hospital death)
- *SPS variables*: SPS information reported in primary care reports, follow-up consultation reports and hospital specialty reports were extensively collected by the researchers and then classified into:
  - *◦ General or systemic SPS*: persistence of fever, fatigue, muscle weakness, musculoskeletal pain, general malaise, oedema and pressure ulcers
  - *◦ Respiratory SPS:* persistent dyspnoea, rib pain, thoracic pain, persistent cough, persistent pharyngeal symptoms
  - *◦ Neurological SPS:* ICU-related polyneuropathy, headache, paraesthesia, movement disturbances, disorientation or confusion, persistent anosmia or dysgeusia
  - *◦ Mental health SPS*: depressive symptoms, anxiety symptoms, sleep disturbances
  - *◦ Haematological SPS*: anaemia, thrombotic manifestations
  - *◦ Dermatological SPS*: pruritus, alopecia, exanthema, eczema
  - *◦ Nephrological SPS*: renal insufficiency de novo
  - *◦ Urological SPS*: dysuria, haematuria, oliguria
  - *◦ Endocrinological SPS*: uncontrolled glycaemia, caloric malnutrition
  - *◦ Otorhinolaryngological SPS*: hypoacusis, otalgia, vertigo symptoms
  - *◦ Ophthalmological SPS*: diplopia, conjunctivitis, visual loss
  - *◦ Digestive SPS*: persistent nausea, vomits, diarrhoea, constipation, anorexia, abdominal pain
  - *◦ Cardiovascular SPS*: syncope or hypotension, arrythmia
  - *◦ Infectious SPS (superinfections after COVID-19 resolution):* urinary tract infections (UTI), pneumonia, mycosis, phlebitis
- *Follow-up outcomes (6 months after discharge):* readmission to Emergency Services, readmission to hospitalization, death

A detailed description and definitions of all these SPS are presented in Additional file 2: Table S1.

### Statistical analyses

A descriptive analysis of the main sociodemographic and clinical variables of the study was conducted, stratified by outcomes (return to Emergency Services, readmission to hospital and death after discharge). The frequency of SPS in surviving patients was described in detail and stratified by sex. Hierarchical cluster analyses of the SPS were made in order to provide evidence of the validity of the information collected and to explore possible unknown associations between different SPS. This analysis was applied to cluster variables rather than observations, using a dissimilarity measure based on Euclidean distances.

Bivariant analyses were then conducted to identify differences in SPS between men and women, and according to the three outcomes. T-test was applied for quantitative variables (mean differences) and chi-squared tests for qualitative variables. When the application conditions were not met, Mann-Whitney and Fisher exact tests were conducted, respectively.

Multiple logistic regression models were applied. The three outcomes were analysed as dependent variables in three models, and SPS were analysed as independent variables. Crude and adjusted odds ratios (cOR and aOR, respectively) were calculated. The models were adjusted for sex, age and sociodemographic and clinical variables, when possible. All statistical analyses were performed using Stata (StataCorp)®, version 15.0.

### Ethical considerations

The ethical implications of the study were considered according to the principles of the Declaration of Helsinki Declaration. The study was approved by the Provincial Research Ethical Committee of Granada on October 1, 2020. As this was a retrospective observational study, the informed consent was not possible. The database was anonymized, and no identification data was used in the analyses.

## Results

### Sociodemographic and clinical variables

Table 1 shows the distribution of the main sociodemographic variables recorded in the study, stratified by outcome. Of the total cohort (*n* = 969), the subgroup of patients who were discharged alive (*n* = 797) constituted the sample followed. The rest (17.8%) represented in-hospital mortality from COVID-19 in our cohort. The 8 patients who died after discharge showed older age (82.1 years) and higher frequency of comorbidities and dependence.

**Table 1:** Sociodemographic and clinical variables of the followed cohort (*n* = 797) stratified for outcomes of the study

Variable	Total sample followed (***n*** = 797)	Return to Emergency Services (***n*** = 160)	Readmission to hospital (***n*** = 35)	Death after discharge (***n*** = 8)
	N (%), x (s)	N (%), x (s)	N (%), x (s)	N (%), x (s)
Men	428 (53.7)	85 (53.1)	15 (42.9)	3 (37.5)
Age: x (s)	63.0 (14.4)	61.7 (15.3)	67.5 (16.9)	82.1 (4.2)
Age categories				
<40 years	45 (5.6)	11 (6.9)	2 (5.7)	0 (0)
40–50 years	93 (11.7)	20 (12.5)	0 (0)	0 (0)
50–60 years	181 (22.7)	39 (24.4)	8 (22.9)	0 (0)
60–70 years	206 (25.8)	43 (26.9)	10 (28.6)	0 (0)
70–80 years	164 (20.6)	25 (15.6)	6 (17.1)	2 (25.0)
80–90 years	95 (11.9)	16 (10.0)	5 (14.3)	6 (75.0)
>90 years	13 (1.6)	6 (3.8)	4 (11.4)	0 (0)
Setting				
Granada	361 (45.3)	75 (46.9)	18 (51.4)	4 (50.0)
Córdoba	186 (23.3)	40 (25.0)	6 (17.1)	1 (12.5)
Cádiz	27 (3.4)	5 (3.1)	0 (0)	0 (0)
Jaén	223 (28.0)	40 (25.0)	11 (31.4)	3 (37.5)
Any comorbidity	538 (67.5)	104 (65.0)	23 (65.7)	7 (87.5)
Hypertension	409 (51.3)	77 (48.1)	18 (51.4)	6 (75.0)
Diabetes mellitus	166 (20.8)	35 (21.9)	7 (20.0)	1 (12.5)
Cardiovascular disease	164 (20.6)	40 (25.0)	13 (37.1)	3 (37.5)
Pneumopathy	107 (13.4)	22 (13.8)	3 (8.6)	2 (25.0)
COPD	40 (5.0)	7 (4.4)	1 (2.9)	1 (12.5)
Asthma	59 (7.4)	11 (6.9)	1 (2.9)	0 (0)
Chronic kidney disease	69 (8.7)	14 (8.8)	5 (14.3)	3 (37.5)
Autoimmune disease	61 (7.7)	17 (10.6)	2 (5.7)	1 (12.5)
Immunosuppression	36 (4.5)	10 (6.3)	2 (5.7)	0 (0)
Active neoplasm	32 (4.0)	6 (3.8)	1 (2.9)	1 (12.5)
Polymedication^a^	295 (37.0)	68 (42.5)	20 (57.1)	6 (75.0)
Dependence	120 (15.1)	29 (18.1)	14 (40.0)	5 (62.5)
Living at home	712 (89.3)	138 (86.3)	24 (68.7)	5 (62.5)
Hospitalization days x (s)	15.0 (13.5)	16.6 (13.8)	22.3 (20.0)	19.4 (12.3)
Ferritin at admission x (s)	685.0 (952.2)	537.1 (546.5)	378.8 (308.5)	372.8 (502.9)
Unknown	108 (13.6)	23 (14.4)	4 (11.4)	1 (12.5)
Abnormal TR at admission	667 (83.7)	128 (80.0)	23 (65.7)	7 (87.5)
Unknown	36 (4.5)	8 (5.0)	3 (8.6)	0 (0)
CRS	298 (37.4)	55 (34.4)	11 (31.4)	2 (25.0)
Unknown	62 (7.8)	13 (8.1)	3 (8.6)	0 (0)
Concomitant infection^b^	111 (13.9)	29 (18.1)	9 (25.7)	1 (12.5)
CURB-65				
0	221 (27.7)	45 (28.1)	4 (11.4)	0 (0)
1	241 (30.2)	44 (27.5)	9 (25.7)	1 (12.5)
2	130 (16.3)	28 (17.5)	10 (28.6)	5 (62.5)
3	21 (2.6)	6 (3.8)	1 (2.9)	0 (0)
4	2 (0.3)	0 (0)	0 (0)	0 (0)
Unknown	182 (22.8)	37 (23.1)	11 (31.4)	2 (25.0)
Not candidate for CPR	138 (17.3)	31 (19.4)	11 (31.4)	5 (62.5)
Unknown	221 (27.7)	37 (23.1)	11 (31.4)	2 (25.0)
Hydroxychloroquine	704 (88.3)	137 (85.6)	26 (74.3)	5 (62.5)
Lopinavir/ritonavir	488 (61.2)	94 (58.8)	21 (60.0)	1 (12.5)
Azithromycin	603 (65.7)	117 (73.1)	24 (68.6)	4 (50.0)
Corticosteroid boluses	285 (35.8)	60 (37.5)	11 (31.4)	3 (37.5)
Tocilizumab	82 (10.3)	18 (11.3)	6 (17.1)	1 (12.5)
Antibiotics	477 (59.8)	99 (61.9)	21 (60.0)	3 (37.5)
ACEI/ARA-II	221 (27.7)	43 (26.9)	12 (34.3)	4 (50.0)
ICU admission	81 (10.8)	16 (10.0)	3 (8.6)	0 (0)
**Total**	**797 (100)**	**160 (20.1)**	**35 (4.4)**	**8 (1.0)**

*ACEI/ARA-II* angiotensin-converting enzyme inhibitor/angiotensin II receptor antagonists, *COPD* chronic obstructive pulmonary disease, *CRS* cytokine release syndrome, *CURB-65* prognostic score based on confusion, blood urea, respiratory rate, blood pressure and age, *ICU* intensive care unit, *TR* thorax radiography. Missing values are presented as “unknown”. Data are presented as mean (x) and standard deviation (s) for quantitative variables and absolute frequency (n) and relative frequency (%) for qualitative variables. ^a^Polymedication was considered when the patient was prescribed 6 or more drugs prior to hospitalization. ^b^Concomitant infection was considered when, during hospitalization, other infectious diseases different from COVID-19 was identified

### Frequency of SPS during the 6-month follow-up

Of the cohort followed, 509 patients (63.9%) presented any SPS during the follow-up. The prevalence of the different SPS, classified by organs and systems, is presented in Table 2. It can be seen that the frequency of SPS was high and varied. The most frequent groups of SPS were respiratory (42.0%), systemic or general (36.1%), digestive (26.2%), neurological (20.8%), mental health (12.2%), dermatological (9.3%), infectious (7.9%), cardiovascular (5.8%), ophthalmological (4.6%), nephrological (4.5%), haematological (4.4%) and urological (4.3%).

**Table 2:** Frequency of sequelae or persistent symptomatology (SPS) during the 6 months after hospital discharge in surviving patients (*n* = 797) stratified by sex

Sequelae or persistent symptoms (SPS)	Total (***n*** = 797)	Men (***n*** = 428)	Women (***n*** = 369)	***P***-value
	N (%), x (s)	N (%), x (s)	N (%), x (s)	
**Any SPS**	509 (63.9)	267 (62.4)	242 (65.6)	0.348
**Any systemic/general SPS**	288 (36.1)	140 (32.7)	148 (40.1)	0.030*
Persistent fever	56 (7.0)	28 (6.5)	28 (7.6)	0.565
Fatigue	176 (22.1)	81 (18.9)	95 (25.7)	0.021*
Muscle weakness	30 (3.8)	20 (4.7)	10 (2.7)	0.147
Musculoskeletal pain	122 (15.3)	53 (12.4)	69 (18.7)	0.014*
General malaise	34 (4.3)	18 (4.2)	16 (4.3)	0.928
Oedema	23 (2.9)	12 (2.8)	11 (3.0)	0.881
Pressure ulcers	14 (1.8)	6 (1.4)	8 (2.2)	0.412
**Any respiratory SPS**	335 (42.0)	183 (42.8)	152 (41.2)	0.655
Dyspnoea	223 (28.0)	128 (29.9)	95 (25.7)	0.192
Rib pain	36 (4.5)	17 (4.0)	19 (5.1)	0.425
Thoracic pain	53 (6.6)	28 (6.5)	25 (6.8)	0.895
Persistent cough	153 (19.2)	77 (20.9)	76 (17.8)	0.266
Persistent pharyngeal symptoms	67 (8.4)	33 (7.7)	34 (9.2)	0.446
**Any neurological SPS**	166 (20.8)	84 (19.6)	82 (22.2)	0.368
ICU-related polyneuropathy^a^	25 (3.1)	16 (3.7)	9 (2.4)	0.294
Headache	42 (5.3)	14 (3.3)	28 (7.6)	0.007*
Paraesthesia	27 (3.4)	18 (4.2)	9 (2.4)	0.169
Movement disturbances	27 (3.4)	13 (3.0)	14 (3.8)	0.556
Disorientation or confusion	21 (2.6)	11 (2.6)	10 (2.7)	0.902
Persistent anosmia or dysgeusia	57 (7.2)	30 (7.0)	27 (7.3)	0.987
**Any mental health SPS**	97 (12.2)	39 (9.1)	58 (15.7)	0.004*
Depressive symptoms	35 (4.4)	11 (2.6)	24 (6.5)	0.007*
Anxiety symptoms	54 (6.8)	18 (4.2)	36 (9.8)	0.002*
Sleep disturbances	39 (4.9)	22 (5.1)	17 (4.6)	0.728
**Any haematological SPS**	35 (4.4)	15 (3.5)	20 (5.4)	0.188
Thrombotic manifestations	25 (3.1)	9 (2.1)	16 (4.3)	0.071
**Any dermatological SPS**	74 (9.3)	34 (7.9)	40 (10.8)	0.160
Pruritus	20 (2.5)	11 (2.6)	9 (2.4)	0.906
Alopecia	24 (3.0)	1 (0.2)	23 (6.2)	<0.001*
Exanthema	25 (3.1)	17 (4.0)	8 (2.2)	0.145
Eczema	12 (1.5)	8 (1.9)	4 (1.1)	0.364
**Any nephrological SPS**	36 (4.5)	18 (4.2)	18 (4.9)	0.649
Renal insufficiency de novo	7 (0.9)	4 (0.9)	3 (0.8)	0.854
**Any urological SPS**	34 (4.3)	21 (4.9)	13 (3.4)	0.335
**Any endocrinological SPS**	12 (1.5)	10 (2.3)	2 (0.5)	0.038*
Glycaemia uncontrol	7 (0.9)	6 (1.4)	1 (0.3)	0.088
**Any otorhinolaryngological SPS**	25 (3.1)	8 (1.9)	17 (4.6)	0.027*
Vertigo symptoms	15 (1.9)	3 (0.7)	12 (3.3)	0.008*
Otoacoustic symptoms	10 (1.3)	4 (0.9)	6 (1.6)	0.382
**Ophthalmological SPS**	37 (4.6)	16 (3.7)	21 (5.7)	0.191
**Digestive symptoms**	129 (26.2)	63 (14.7)	66 (17.9)	0.226
Diarrhoea	82 (10.3)	35 (8.2)	47 (12.7)	0.035*
Constipation	14 (1.8)	7 (1.6)	7 (1.9)	0.779
Vomiting	16 (2.0)	3 (0.7)	13 (3.5)	0.005*
Abdominal pain	43 (5.4)	20 (4.7)	23 (6.2)	0.331
Anorexia	8 (1.0)	4 (0.9)	4 (1.1)	0.833
**Cardiovascular SPS**	46 (5.8)	26 (6.1)	20 (5.4)	0.693
Hypotension or syncope	23 (2.9)	14 (3.3)	9 (2.4)	0.484
Arrythmia or palpitations	25 (3.1)	13 (3.0)	12 (3.3)	0.862
**Superinfection**	63 (7.9)	30 (7.0)	33 (8.9)	0.313
Urinary tract infection	31 (3.9)	15 (3.5)	16 (4.3)	0.545
Pneumonia	6 (0.8)	4 (0.9)	2 (0.5)	0.691
Mycosis	11 (1.4)	6 (1.4)	5 (1.4)	0.955
**Return to Emergency Services**	160 (20.3)	85 (20.0)	75 (20.7)	0.818
**Readmission to hospital**^b^	35 (12.1)	15 (9.9)	20 (14.5)	0.227
**Death after discharge**	8 (1.0)	3 (0.7)	5 (1.4)	0.356
**Total**	**797 (100)**	**428 (53.7)**	**369 (46.3)**	**–**

*ICU* intensive care unit. The SPS presented in < 5 patients (prevalence < 0.6%) were not considered in the analyses to avoid saturation of information and facilitate the comprehension of the table. ^a^Currently, it is preferred to use the term “muscular debility acquired in ICU”. ^b^Data referred to the subgroup of patients that returned to Emergency Services (*n* = 160). *P < 0.05 of chi-squared tests comparing sex and each SPS

**Fig. 1:**
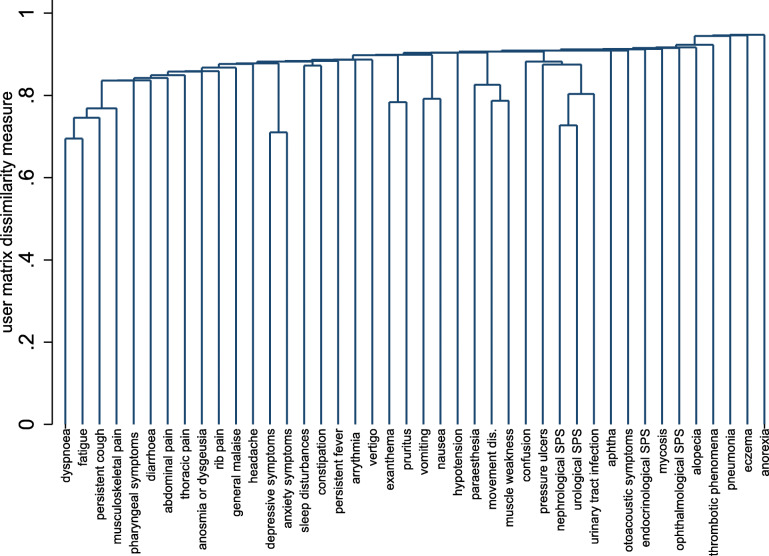
Dendrogram for cluster analysis of sequelae and persistent symptomatology (SPS). Links between vertical lines in the dendrogram link variables or groups of variables with lower dissimilarities than those marked in the vertical axis of the graph. Therefore, lower links between variables indicate stronger relationships between them

In order to provide evidence of the validity of the information collected, a cluster analysis was performed for all SPS (Fig. 1). The dendrogram for specific SPS showed an association between dyspnoea and fatigue, depressive and anxiety symptoms, exanthema and pruritus, vomiting and nausea, nephrological and urological SPS, and both with urinary tract infection. Figure 1 shows the SPS associations in the vertical (ordered) edge: the stronger the association between the SPS, the lower the line linking them. Therefore, “vomiting” and “nausea” should be clearly associated if the data have been properly collected, and the same for the other logical associations shown. Therefore, these cluster associations support the validity of the data collected.

### Outcomes

We analysed outcomes after 6 months of follow-up in the patients who survived the first COVID-19 hospitalization (*n* = 797): return to Emergency Services, hospital readmission and death after discharge. The main SPS associated with the outcomes in the bivariant analyses are shown in Additional file 2: Table S2. There were several SPS that showed association (*P* < 0.05) with all outcomes analysed, such as persistent fever, pressure ulcers, headache, nephrological SPS, hypotension or syncope and superinfection, especially pneumonia (see Additional file 2 for full details).

The main factors associated with return to the Emergency Services (*n* = 160) in the adjusted logistic regression models were persistent fever, thoracic pain, dermatological SPS, arrythmia or palpitations, superinfection and pneumonia (Additional file 2: Table S3).

For hospital readmissions (*n* = 35), the main SPS associated with this outcome in our analysis were persistent fever, any nephrological SPS, superinfection and pneumonia. However, we observed large confidence intervals indicating unstable standard errors (Additional file 2: Table S4).

As only 8 post-discharge deaths were observed, no associations were calculated for this outcome due to limited external validity. The main sociodemographic or clinical factor associated with mortality after discharge was older age. After adjusting for age, most associations disappeared (Additional file 2: Table S5).

**Fig. 2:**
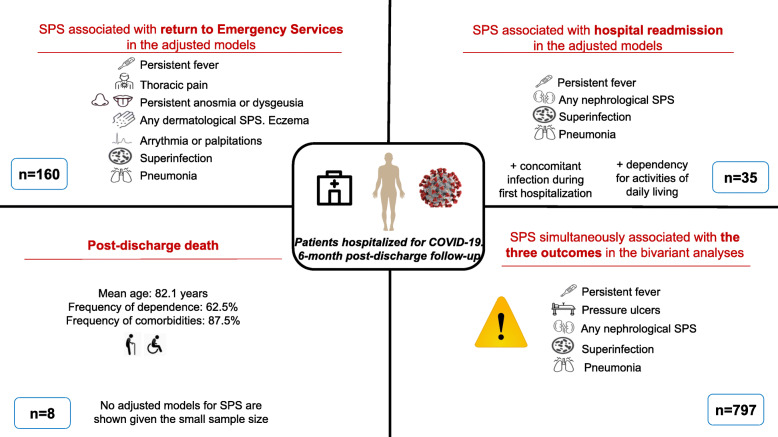
Sequelae and persistent symptomatology (SPS) associated with 6-month follow-up outcomes

The main SPS associated with the outcomes studied in the adjusted models are summarized and illustrated in Fig. 2.

## Discussion

In this study, we described the main characteristics of discharged patients (*n* = 797) from a cohort of 969 patients who required hospitalization during the first months of the COVID-19 pandemic in four hospitals in southern Spain. We provided a detailed list of SPS during the 6 months after discharge and described which of these SPS are associated with negative outcomes (return to Emergency Services, hospital readmission and post-discharge death).

### Characteristics of the cohort

The characteristics of our sample are consistent with other cohorts of patients hospitalized during the first wave of the COVID-19 pandemic in different countries. Therefore, the mean age of our sample (63.0) and the sex distribution (53.7% of men) are similar to previous studies such as the recently published Mediterranean cohort study [18], studies conducted in Italy [21] and Brazil [22] and other Spanish multicentre studies [23], although slightly different from other national reports [24]. For example, Carfi et al. showed 56.5 mean age and 63% of men [20] and Garrigues et al. showed 58 mean age and 63% of women [12].

Our higher mean age could be partially explained by the high life expectancy in our country and by an ageing reference population in South Spain [25]. Also, during the first weeks of the pandemic in Spain, a high number of elderly patients from residential care homes were hospitalized [26], given that exhaustive prevention and control measures had not yet been established. The in-hospital mortality of our cohort was 17.8%, data similar to those reported in other studies in the same period performed in New York [27] but lower than in other studies performed in Wuhan [28] and Spain [23]. Similarly, the proportion of patients requiring intensive care was 12.1%, which is consistent with large patient cohorts in the USA (14.2%) [27].

Since the sample of our study is composed of the most severe COVID-19 cases (those requiring hospitalization), they presented a high frequency of previous diseases, polymedication and dependency for daily living activities, as shown in Table 1.

### Frequency of SPS

We showed 63.9% of SPS during the 6 months after discharge, similar to other studies. A study conducted in Spain with 14 weeks of follow-up showed 50% post-acute COVID-19 syndrome [18]. Another study conducted in France showed 55% persistent symptoms after a mean of 110 days of follow-up [12] and 62.5% of SPS was present 50 days after discharge [19]. However, other series have reported up to 68% at 2 months [29], 87% at 2 months [20] and 87% at 3 months [17].

Table 2 lists all reported SPS and their prevalence stratified by sex. The most relevant were as follows.

#### Respiratory SPS

Dyspnoea was the most frequent specific SPS in our cohort (28.0%). These data are lower than other reported frequencies, 31.4% [19] and 43.4% [20]. All reported series concur that dyspnoea and fatigue are the most frequent SPS after COVID-19 [12, 18, 20, 30]. A possible explanation for these symptoms could be the persistence of fibrotic residual pulmonary areas. Furthermore, fibrosis would be the result of an ineffective organization stage after the initial acute inflammatory response [31]. Thoracic pain (6.6%) was associated with return to the Emergency Services. Therefore, the presence of this symptom could warn of potential severity.

#### Systemic SPS

The high prevalence of fatigue (22.1%) is consistent with previous series [20, 30, 32]. A high frequency of musculoskeletal pain (15.3%) was also observed. Both symptoms could be explained by the systemic inflammatory response generated by COVID-19 [33, 34] and by natural recovery after hospitalization processes [34]. Persistent fever (7.0%) and pressure ulcers (1.8%), generally occurring in patients with longer hospitalization time, were associated with negative outcomes. Therefore, the presence of these signs could be related to particularly vulnerable patients that require more intense monitoring.

#### Digestive SPS

Digestive SPS were very frequent (26.2%), especially diarrhoea (10.3%) and abdominal pain (5.4%), according to previous series [20]. Some digestive SPS, such as vomiting (2.0% in our cohort), diarrhoea, nausea, hepatitis or abdominal pain, have been associated with COVID-19 treatment, as adverse drug reactions [35]. According to other studies, hepatic injuries may also be associated with COVID-19 [36].

#### Neurological SPS

Several recent studies pointed the high frequency of neurological SPS after COVID-19 [37, 38]. In our cohort (20.8%), the most prevalent SPS was persistent anosmia or dysgeusia (7.2%), which showed a protective association with negative outcomes. Headache (5.3%) was associated with the outcomes, and disorientation or confusion (2.6%) was associated with hospital readmission. The presence of these symptoms could predict a worse evolution and, therefore, require timely preventive measures. Finally, the high prevalence of paraesthesia (3.4%) and movement (3.4%) disorders, such as dystonia or tremor, was surprising. These SPS were recently pointed out as potentially involved in COVID-19 complications [38]. A wide variety of neurological manifestations (e.g. encephalopathy, encephalitis, seizures, cerebrovascular events, acute polyneuropathy, etc.) have been associated with COVID-19 [39], some of them also confirmed at the neuropathological level [40]. Apart from symptoms, COVID-19 has also been linked to a variety of severe neurological complications, especially Guillain-Barré syndrome [41].

#### Mental health SPS

The high frequency of mental health symptoms in our cohort (12.2%) reflects the alarm expressed by several authors [42, 43] on the importance of preventing and identifying mental health SPS after hospitalization for COVID-19. These symptoms could be overestimated due to isolation during the hospitalization period and lockdown measures during the first wave of the pandemic [44, 45], but also due to other simultaneous familiar cases and admissions as well as high uncertainty during the first months. We found a high frequency of anxiety (6.8%), sleep disturbances (4.9%) and depressive symptoms (4.4%), especially higher in women.

#### Dermatological SPS

This group represented 9.3% of our cohort. Although a high prevalence of dermatological symptoms related to COVID-19 has been reported since the beginning of the pandemic [46], we did not find any studies reporting these frequencies in follow-up cohorts. In our study, these symptoms were associated with return to the Emergency Services, possibly because they are visible and worrying to patients, but we found no association with mortality or hospital readmission. The most frequent SPS in our study was exanthema (3.1%). The high presence of alopecia (3.0%) in our study, especially higher in women, is noteworthy.

#### Superinfection

One of the most intriguing findings of our study was the high prevalence of superinfection (7.9%) during the 6 months after discharge from COVID-19 and its strong association with negative outcomes. It is possible that COVID-19 produces medium-term immunosuppression leading to superinfection, especially pneumonia, as reported in other works [33, 34]. This is consistent with other viral infections such as influenza, especially bacterial respiratory superinfections. The most frequent SPS in our cohort were urinary tract infection (3.9%) and mycosis (1.4%), which were not associated with negative outcomes. However, the presence of pneumonia (0.8%) led to negative outcomes in the adjusted analyses, thus becoming one of the main alarm symptoms proposed in our study. We were able to identify *Streptococcus pneumoniae* as one of the agents involved in 2 of the 6 cases of pneumonia, but no other etiological diagnosis was available.

#### Cardiovascular SPS

Several authors have reported the potential cardiovascular effects of COVID-19 [47, 48], as occur with other systemic viral infections like influenza [49]. Longitudinal studies reported frequencies of cardiovascular SPS higher than 10% [50]. In our sample, 5.8% of patients reported some cardiovascular SPS. Specifically, arrythmia or palpitations (3.1%) and hypotension or syncope (2.9%) were also associated with negative outcomes.

#### Ophthalmological SPS

We present the highest frequency of reported ophthalmological SPS in a cohort of hospitalized patients (4.6%), including conjunctivitis and vision loss, among others. Although not associated with negative outcomes, our results suggest that COVID-19 may be implicated in ophthalmic outcomes, as noted by other authors [51].

#### Nephrological SPS

In our cohort, 4.5% of patients presented nephrological SPS and 0.9% showed de novo renal insufficiency. It is possible that some nephrological SPS might be related to the nephrotoxicity of COVID-19 treatment. However, this has been mainly associated with remdesivir [52], which was not involved in therapeutic approaches in our cohort during the first wave of the pandemic. The most important aspect of these SPS is their strong association with negative outcomes in our study. Therefore, patients who develop nephrological symptomatology after discharge should be especially monitored, as these SPS could signal a particularly vulnerable patient profile [53].

#### Haematological SPS

In agreement with other studies that reported this relationship very early [54, 55], our study shows thrombotic manifestations (4.4%), namely deep vein thrombosis, acute pulmonary embolism or cerebral stroke as the main haematological SPS in our cohort.

#### Urological SPS

Urological SPS were present in 4.3% of the patients followed. Symptoms of voiding syndrome such as dysuria, oliguria or nocturia, but also haematuria were relatively frequent in our cohort. Although the impact of the pandemic on urological services has been studied [56], these symptoms have not been consistently associated with COVID-19.

#### Otorhinolaryngological SPS

We present 3.1% of otorhinolaryngological SPS. To the best of our knowledge, these are not well-known SPS of COVID-19, although some authors have pointed out their possible implications [57]. We observed 1.9% of vertigo SPS reported (rotatory dizziness, tinnitus, etc.) after discharge. More data are still necessary to draw conclusions about this association.

#### Endocrinological SPS

The last group of SPS identified in our cohort were endocrinological SPS (1.5%), especially uncontrolled glycaemia (0.9%) in diabetic patients. This group was the only one that presented higher frequencies in men.

### Return to the Emergency Services

This outcome has not yet been analysed in depth in previous studies. However, given the increasing number of patients discharged from COVID-19, the healthcare costs and the current saturation of the Emergency Services, it is important to detect which SPS cause patients to return to these services. Although we found a high frequency of patients who returned to the Emergency Services (20.3%), it is possible that these data are even underestimated, given the high concern for seeking healthcare at the onset of the pandemic. In our cohort, it was interesting to find that previous diseases, dependency, living in residential care homes or prognostic scores showed no association with this return. However, visible or easily identifiable symptoms like dermatological SPS and persistent anosmia or dysgeusia (which did not increase the risk of mortality or hospital readmission in our cohort) were strongly associated with return to the Emergency Services. It is possible that these SPS could be appropriately addressed by primary care telephone consultations. SPS that showed association with other negative outcomes, such as thoracic pain, arrhythmia, fever and pneumonia, which are undoubtedly alarming symptoms, were also associated with necessary return to the Emergency Services. Recognition of the severity of COVID-19 symptoms is crucial for correct therapeutic management [11], which should also improve follow-up treatment. We believe that more information on the potential alarming symptoms of patients discharged from COVID-19 could be provided to patients and their caregivers to improve follow-up and preventive measures in primary care.

### Limitations

We present the prevalence of SPS during the 6 months after discharge of patients hospitalized for COVID-19. No distinctions were made between early or long-term SPS and any interpretation of the results of this study should take into account that our exposures are SPS during the 6 months after discharge, at any time point. Since the data assessment was collected retrospectively and no prior standardization of SPS was designed, analytical associations through OR are of limited value and should be interpreted with caution. However, we present these results as potential risk factors to be considered in future longitudinal studies. Also, we cannot confirm that these SPS are a consequence of COVID-19, as there is no available comparison group (prevalence of all SPS in patients that did not suffer from COVID-19). We attempted to minimize this limitation by suggesting only potential risk factors to be taken into account during patient follow-up and analysing their relationship with potential negative outcomes in adjusted models. However, the adjusted models could not be well optimized for two outcomes: hospital readmission and death after discharge, given their small sample size (35 and 8, respectively). Therefore, both models were adjusted for a small number of variables and the validation criteria were not always optimal. Given this limitation, associations on post-discharge death are only presented as Additional file 2: Table S5. In addition, obesity and smoking could not be adequately collected and, therefore, models were not adjusted for these important variables. The aim of this study, however, is to describe SPS and propose (but not corroborate or conclude) possible risk factors on negative outcomes.

Our study is also conducted in a sample of high-risk COVID-19 patients (i.e. those who required hospitalization). Therefore, the results regarding SPS frequencies and association with outcomes could not be extrapolated to all COVID-19 patients, but only to those who required hospital care. Also, the SPS reported by this cohort could be attributable to COVID-19 or could be partially explained by the hospitalization process and the treatment received.

The study was conducted during the first wave of COVID-19, when diagnostic and therapeutic protocols were different from the current ones. This bias was addressed by adjusting all associations for diagnostic and therapeutic variables.

Finally, our study was conducted in Spain. External validity may be biased given that the life expectancy, health care and baseline characteristics of Spanish patients cannot be extrapolated to other countries. We tried to minimize this effect by designing a multicentre study including 4 different hospitals and 969 patients. The results of this study could be applied to the Spanish population but should be included in systematic reviews and compared with international samples to optimize accurate conclusions.

## Conclusions

Patients who required hospitalization for COVID-19 during the first wave in Spain presented a high frequency of SPS (63.9%) during the first 6 months after discharge. We presented a detailed list of SPS and their frequency, stratified by sex. The most frequent groups were respiratory, systemic, neurological, mental health, superinfection and dermatological SPS. We also presented the association of these SPS with negative outcomes (return to the Emergency Services, hospital readmission and death after discharge). In sum, the presence of persistent fever, pneumonia, nephrological SPS and pressure ulcers indicated a high-risk patient profile.

Follow-up strategies should be optimized to avoid negative outcomes and to individualize preventive measures from primary care follow-up in subsequent waves.

## Supplementary Information

**Additional file 1:** STROBE checklist. Checklist of items included in reports of observational studies*.*

**Additional file 2: Tables S1 to S5** (detailed information on secondary statistical results and data collection). **Table S1.** Sequelae or persistent symptoms (SPS) during 6 months after discharge: description and definitions of the information collected. **Table S2.** Sequelae or persistent symptoms (SPS) during 6 months after discharge associated to follow-up outcomes: return to emergency care, hospital readmission and post-discharge death. **Table S3.** Factors associated with the return to Emergency Services during the 6 months after discharge for COVID-19 hospitalization (*n*=160). Crude and adjusted logistic regression models. **Table S4.** Factors associated with hospital readmission during the 6 months after discharge for COVID-19 first hospitalization (*n*=35). Crude and adjusted logistic regression models. **Table S5.** Factors associated with mortality during the 6 months after discharge for COVID-19 hospitalization (*n*=8). Crude and adjusted logistic regression models.

## Data Availability

Data described in the manuscript and the analytical code will be made available during ANCOHORT study conduct only to the investigators who have participated in/contributed to the study. Select summary data may be shared with policy makers for specific purposes. The study executive will consider specific requests for data analyses by non-contributing individuals upon reasonable request to the corresponding author.
