# Supplementary material for: Sequelae, persistent symptomatology and outcomes after COVID-19 hospitalization: the ANCOHVID multicentre 6-month follow-up study
Source: BMC Med. 2021 May 20;19:129. doi: 10.1186/s12916-021-02003-7 (PMC8134820; doi:10.1186/s12916-021-02003-7)
Supplement: Supplementary file 1 — Additional file 1: STROBE checklist. Checklist of items included in reports of observational studies. [file 12916_2021_2003_MOESM1_ESM.pdf]

Additional File 1. Sequelae, persistent symptomatology, and outcomes after COVID-19 hospitalization: the ANCOHVID multicentre 6-month follow-up study.

Álvaro Romero-Duarte, Mario Rivera-Izquierdo, Inmaculada Guerrero-Fernández de Alba, Marina Pérez-Contreras, Nicolás Francisco Fernández-Martínez, Rafael Ruiz-Montero, Álvaro Serrano-Ortiz, Rocío Ortiz González-Serna, Inmaculada Salcedo-Leal, Eladio Jiménez-Mejías,[3,9] Antonio Cárdenas-Cruz.

STROBE Statement

Checklist of items included in reports of observational studies.

	Item No	Recommendation
**Title and abstract**	1	(*a*) Indicate the study's design with a commonly used term in the title or the abstract. **Retrospective observational longitudinal (6-month follow-up) study (abstract).**
		(*b*) Provide in the abstract an informative and balanced summary of what was done and what was found. **Provided in the abstract**.
**Introduction**		
Background/rationale	2	Explain the scientific background and rationale for the investigation being reported. **Explained in the first 4 paragraphs of the introduction.**
Objectives	3	State specific objectives, including any prespecified hypotheses. **Stated in the last (fifth) paragraph of the introduction.**
**Methods**		
Study design	4	Present key elements of study design early in the paper. **Presented at the beginning of the methods (study design and setting).**
Setting	5	Describe the setting, locations, and relevant dates, including periods of recruitment, exposure, follow-up, and data collection. **Described at the beginning of the methods (study design and setting).**
Participants	6	Give the eligibility criteria, and the sources and methods of selection of participants. **Presented at the beginning of the methods (study design and setting).**
Variables	7	Clearly define all outcomes, exposures, predictors, potential confounders, and effect modifiers. Give diagnostic criteria, if applicable. **We presented all the variables of the study in the "data source and variables" section. We indicated the exposure and outcome variables.**
Data sources/ measurement	8*	For each variable of interest, give sources of data and details of methods of assessment (measurement). Describe comparability of assessment methods if there is more than one group. **We presented all the sources of the variables of the study in the "data source and variables" section and provided a full description in**

		**Additional File 2: Table S1. We also performed a dendrogram for cluster analysis (figure 1) in order to provide evidence of validity of the information collected.**
Bias	9	Describe any efforts to address potential sources of bias. **Described in "statistical analyses" section.**
Study size	10	Explain how the study size was arrived at. **Explained in the methods (a complete cohort of hospitalized patients during the period of recruitment).**
Quantitative variables	11	Explain how quantitative variables were handled in the analyses. If applicable, describe which groupings were chosen and why. **Explained in the methods.**
Statistical methods	12	(*a*) Describe all statistical methods, including those used to control for confounding. **Described in "statistical analyses" section.**
		(*b*) Describe any methods used to examine subgroups and interactions. **Described in "statistical analyses" section.**
		(*c*) Explain how missing data were addressed. **Explained throughout the manuscript (two variables were not considered for presenting too many missing values: smoking habit and obesity).**
		If applicable, describe analytical methods taking account of sampling strategy. **N/A.**
		(*e*) Describe any sensitivity analyses. **N/A.**

**Results**		
Participants	13*	(a) Report numbers of individuals at each stage of study—eg numbers potentially eligible, examined for eligibility, confirmed eligible, included in the study, completing follow-up, and analysed. **Explained in the first paragraph of the "Sociodemographic and clinical variables" section.**
		(b) Give reasons for non-participation at each stage. **We included all hospitalized patients during recruitment (non-participation is not applicable in this study).**
		(c) Consider use of a flow diagram. **N/A**
Descriptive data	14*	(a) Give characteristics of study participants (eg demographic, clinical, social) and information on exposures and potential confounders. **Explained in the first paragraph of the "Sociodemographic and clinical variables" section and in Table 1.**
		(b) Indicate number of participants with missing data for each variable of interest. **Indicated in Table 1 ("Unknown").**
Outcome data	15*	*Cross-sectional study*—Report numbers of outcome events or summary measures. **Reported in Table 1 (columns).**
Main results	16	(*a*) Give unadjusted estimates and, if applicable, confounder-adjusted estimates and their precision (eg, 95% confidence interval). Make clear which confounders were adjusted for and why they were included. **Given in Additional File 2: Tables S3 to S5.**

		(*b*) Report category boundaries when continuous variables were categorized. **Reported in Table 1 (age).**
		(*c*) If relevant, consider translating estimates of relative risk into absolute risk for a meaningful time period. **N/A**
Other analyses	17	Report other analyses done—eg analyses of subgroups and interactions, and sensitivity analyses. **Reported in the “Frequency of SPS during the 6-month follow-up” and “Outcomes” sections.**
**Discussion**		
Key results	18	Summarise key results with reference to study objectives. **Reported in the first paragraph of the Discussion.**
Limitations	19	Discuss limitations of the study, taking into account sources of potential bias or imprecision. Discuss both direction and magnitude of any potential bias. **Thoroughly reported in the Limitations subsection.**
Interpretation	20	Give a cautious overall interpretation of results considering objectives, limitations, multiplicity of analyses, results from similar studies, and other relevant evidence. **Reported throughout the Discussion and Conclusion sections.**
Generalisability	21	Discuss the generalisability (external validity) of the study results. **Reported in the Limitations subsection**
**Other information**		
Funding	22	Give the source of funding and the role of the funders for the present study and, if applicable, for the original study on which the present article is based. **Reported in the “Funding” declaration.**
