# Supplementary material for: Sequelae, persistent symptomatology and outcomes after COVID-19 hospitalization: the ANCOHVID multicentre 6-month follow-up study
Source: BMC Med. 2021 May 20;19:129. doi: 10.1186/s12916-021-02003-7 (PMC8134820; doi:10.1186/s12916-021-02003-7)
Supplement: Supplementary file 2 — Additional file 2: Tables S1 to S5 (detailed information on secondary statistical results and data collection). Table S1. Sequelae or persistent symptoms (SPS) during 6 months after discharge: description and definitions of the information collected. Table S2. Sequelae or persistent symptoms (SPS) during 6 months after discharge associated to follow-up outcomes: return to emergency care, hospital readmission and post-discharge death. Table S3. Factors associated with the return to Emergency Services during the 6 months after discharge for COVID-19 hospitalization (n=160). Crude and adjusted logistic regression models. Table S4. Factors associated with hospital readmission during the 6 months after discharge for COVID-19 first hospitalization (n=35). Crude and adjusted logistic regression models. Table S5. Factors associated with mortality during the 6 months after discharge for COVID-19 hospitalization (n=8). Crude and adjusted logistic regression models. [file 12916_2021_2003_MOESM2_ESM.pdf]

# Additional File 2. Sequelae, persistent symptomatology, and outcomes after COVID-19 hospitalization: the ANCOHVID multicentre 6-month follow-up study.

**Álvaro Romero-Duarte, Mario Rivera-Izquierdo, Inmaculada Guerrero-Fernández de Alba, Marina Pérez-Contreras, Nicolás Francisco Fernández-Martínez, Rafael Ruiz-Montero, Álvaro Serrano-Ortiz, Rocío Ortiz González-Serna, Inmaculada Salcedo-Leal, Eladio Jiménez-Mejías,[3,9] Antonio Cárdenas-Cruz.**

**Table S1. Sequelae or persistent symptoms (SPS) during 6 months after discharge: description and definitions of the information collected.**

| Sequelae or persistent symptoms (SPS) | Definition |
|---:|:---:|
| **Any SPS** | Any of the considered SPS were present at any point during the follow-up. |
| **Any systemic/general SPS** | Any of the following SPS were present at any point during the follow-up: |
| Persistent fever | Temperature >38ºC measured in Primary Care consultation or reported by the patient during the follow-up |
| Fatigue* | Subjective sensation of physical tiredness. |
| Muscle weakness* | Subjective sensation of lack of muscle strength. |
| Musculoskeletal pain | Pain in any muscle or articulation not reported before COVID-19 hospitalization. |
| General malaise | Subjective sensation of unhealthiness, discomfort or weakness that cannot be attributed to any specific condition. |
| Edema | Objective dimple sign in Primary Care consultation or increasing swelling reported by the patient |
| Pressure ulcers | Ulcers in sloping areas after prolonged bedtime, not present before COVID-19 hospitalization |
| **Any respiratory SPS** | Any of the following SPS were present at any point during the follow-up: |
| Dyspnea | Subjective sensation of difficult or labored breathing that was not present (or was less significant) before COVID-19 hospitalization |
| Rib pain | Muscle or bone pain in the rib |
| Thoracic pain | Pain in the thoracic area |
| Persistent cough | Cough identified on inspection at Primary Care consultation or reported after discharge from COVID-19 that was not present or was less significant before COVID-19 hospitalization |
| Persistent pharyngeal symptoms | Odynophagia, pharyngeal discomfort or sore throat that was not present before COVID-19 hospitalization |
| **Any neurological SPS** | Any of the following SPS were present at any point during the follow-up: |
| ICU-related polyneuropathy | Polyneuropathy associated with ICU hospitalization. |
| Headache | Any type of headache in patients that do not suffer from chronic headache (e.g., migraine) |
| Paresthesia | Subjective sensation of tingle or lack of sensitivity in any part of the body |

| | |
|---|---|
| Movement disturbances | Shaking, tremor or dystonia that were not present before COVID-19 hospitalization |
| Disorientation or confusion | Subjective sensation reported by the patient or caregiver of bouts of confusion |
| Persistent anosmia or dysgeusia | Anosmia or dysgeusia reported by the patient after discharge |
| **Any mental health SPS** | Any of the following SPS were present at any point during the follow-up: |
| Depressive symptoms* | Sadness, apathy, anodyne or hopelessness symptoms reported by the patient but not measured in quantitative scales |
| Anxiety symptoms* | Anxiety, restlessness or extreme fear reported by the patient but not measured in quantitative scales |
| Sleep disturbances | Insomnia or lack of rest reported by the patient and not present before COVID-19 hospitalization |
| **Any hematological SPS** | Disturbances in the blood count reported during follow-up consultations or presence of the following SPS: |
| Thrombotic phenomena | Deep vein thrombosis, acute pulmonary embolism, stroke. |
| **Any dermatological SPS** | Any of the following SPS were present at any point during the follow-up: |
| Pruritus | Generalized itching or itching in specific parts of the body that was not present before COVID-19 hospitalization |
| Alopecia | Hair loss that was not present before COVID-19 hospitalization |
| Exanthema | Exanthema lesions or generalized skin rash that were not present before COVID-19 hospitalization |
| Eczema | Eczematous lesions or intertrigo that were not present before COVID-19 hospitalization |
| **Any nephrological SPS** | Any of the following SPS were present at any point during the follow-up: |
| Renal insufficiency *de novo* | Analytical objective renal failure signs (urea, creatinine, etc.), that were not present or were less severe before COVID-19 hospitalization. |
| **Any urological SPS*** | Voiding syndrome symptoms reported by the patients (dysuria, nocturia, hematuria, etc.). |
| **Any endocrinological SPS** | Any of the following SPS were present at any point during the follow-up: |
| Uncontrolled glycemia | Objective measurement of glycemia out of normal parameters. |
| **Any otorhinolaryngological SPS** | Any of the following SPS were present at any point during the follow-up: |
| Vertigo symptoms | Vertigo, rotatorydizziness or tinnitus that were not present before COVID-19 hospitalization |
| Otoacoustic symptoms | Hearing loss, otitis or otalgia that were not present before COVID-19 hospitalization |
| **Ophthalmological SPS** | Loss of vision, diplopia or conjunctivitis that were not present before COVID-19 hospitalization |
| **Digestive symptoms*** | Any of the following SPS were present at any point during the follow-up: |
| Diarrhea* | Reported by patients or their caregivers. |
| Constipation* | Reported by patients or their caregivers. |
| Vomiting* | Reported by patients or their caregivers. |
| Abdominal pain* | Pain in the abdominal area. |
| Anorexia* | Loss of appetite. |
| **Cardiovascular SPS** | Any of the following SPS or cardiac insufficiency were present at any point during the follow-up: |
| Hypotension or syncope | Objective measurement of arterial pressure $<90/60$ or reported symptoms of syncope or frequent orthostatic hypotension not present or less severe before COVID-19 hospitalization |

| | |
|---|---|
| Arrythmia or palpitations | Objective identification of arrythmia in electrocardiographic test in consultation or reported symptoms of palpitations that was not present before COVID-19 hospitalization |
| **Superinfection** | New infection by a pathogen different from SARS-CoV-2 after discharge: |
| Urinary tract infection | Pathogen detected in urinary culture |
| Pneumonia | Pathogen detected in respiratory sample or sputum |
| Mycosis | Fungus identification (mostly Candida albicans) |

*At discharge, most of the SPS were not present (e.g., no fever is a necessary criterion for discharge). However, the SPS that could be present at discharge and continue during follow-up are marked with an asterisk. The rest were not present at discharge and appeared abruptly during the follow-up of the study.

**Table S2. Sequelae or persistent symptoms (SPS) during 6 months after discharge associated to follow-up outcomes: return to emergency care, hospital readmission and post-discharge death.**

| Sequelae or persistent symptoms (SPS) | Return to Emergency Services P-value | Hospital readmission P-value | Post-discharge death P-value |
|---|---|---|---|
| **Any SPS** | 0.004 | 0.301 | 0.224* |
| **Any systemic/general SPS** | 0.003 | 0.001 | 1.000* |
| Persistent fever | <0.001 | <0.001 | 0.046 |
| Fatigue | 0.649 | 0.787* | 0.693* |
| Muscle weakness | 0.178 | 0.628* | 0.265* |
| Musculoskeletal pain | 0.232 | 0.069* | 0.109* |
| General malaise | <0.001 | 0.092* | 0.296* |
| Edema | 0.774 | 1.000* | 0.210* |
| Pressure ulcers | 0.012* | 0.005* | 0.008* |
| **Any respiratory SPS** | 0.001 | 0.001 | 0.291 |
| Dyspnea | 0.005 | 0.005 | 0.229 |
| Rib pain | 0.214 | 1.000* | 1.000* |
| Thoracic pain | <0.001 | 0.563* | 0.669* |
| Persistent cough | 0.882 | 0.071 | 0.711* |
| Persistent pharyngeal symptoms | 0.900 | 0.704* | 0.634* |
| **Any neurological SPS** | 0.172 | 0.369 | 0.041 |
| ICU-related polyneuropathy | 0.969 | 1.000* | 0.226* |
| Headache | 0.031 | 0.012* | 0.012 |
| Paresthesia | 0.028 | 0.371* | 1.000* |
| Movement disturbances | 0.220 | 0.390* | 1.000* |
| Disorientation or confusion | 0.132 | 0.005* | 0.001* |
| Persistent anosmia or dysgeusia | 0.025 | 0.615* | 0.657* |
| **Any mental health SPS** | 0.042 | 0.030* | 0.253* |
| Depressive symptoms | 0.074 | 0.165* | 0.303* |
| Anxiety symptoms | 0.064 | 0.092* | 1.000* |
| Sleep disturbances | 0.096 | 0.044* | 0.054* |
| **Any hematological SPS** | 0.416 | 0.044* | 0.303* |
| Thrombotic phenomena | 0.331 | 0.009* | <0.001 |
| **Any dermatological SPS** | <0.001 | 0.755* | 1.000* |
| Pruritus | 0.010* | 0.606* | 1.000* |
| Alopecia | 0.220 | 0.602* | 1.000* |
| Exanthema | 0.041* | 0.615* | 1.000* |
| Eczema | 0.020* | 0.635* | 1.000* |
| **Any nephrological SPS** | 0.016* | <0.001* | 0.004* |
| Renal insufficiency *de novo* | 0.005* | 0.014* | 1.000* |
| **Any urological SPS** | 0.757 | 0.025* | 0.004 |
| **Any endocrinological SPS** | 0.020* | 1.000* | 1.000* |
| Uncontrolledglycemia | 0.034* | 1.000* | 1.000* |
| **Any otorhinolaryngological SPS** | 0.605* | 1.000* | 0.226* |
| Vertigo symptoms | 0.916* | 1.000* | 1.000* |
| Otoacoustic symptoms | 0.397* | 0.321* | 0.096* |
| **Ophthalmological SPS** | 0.144 | 1.000* | 1.000* |

| | | | |
|---|---|---|---|
| **Digestive symptoms** | 0.008 | 0.014* | 0.622* |
| Diarrhea | 0.331 | 0.013* | 1.000* |
| Constipation | 0.499* | 0.006* | 0.008* |
| Vomiting | 1.000* | 0.039* | 1.000* |
| Abdominal pain | 0.011 | 1.000* | 1.000* |
| Anorexia | 1.000* | 0.227* | 1.000* |
| **Cardiovascular SPS** | 0.153* | 0.604* | 0.201* |
| Hypotension or syncope | 0.033* | 0.081* | 0.020* |
| Arrythmia o palpitations | 0.002* | 0.135* | 0.226* |
| **Superinfection** | 0.001 | <0.001 | 0.020* |
| Urinary tract infection | 0.437 | 0.059* | 0.273* |
| Pneumonia | 0.018* | 0.014* | 0.059* |
| Mycosis | 1.000* | 1.000* | 1.000* |
| **Return to Emergency Services** | - | <0.001 | 0.370* |
| **Readmission to Hospital** | <0.001 | - | 0.002* |
| **Death after discharge** | 0.370* | 0.002* | - |
| **Total** | **160 (20.3)** | **35 (12.1)** | **8 (1.0)** |

*ICU, intensive care unit. P-values are presented for chi-squared test, after checking the conditions of application. * P-value of the Fisher exact test.*

**Table S3. Factors associated with the return to Emergency Services during the 6 months after discharge for COVID-19 hospitalization (n=160). Crude and adjusted logistic regression models.**

| Sequelae or persistent symptoms (SPS) | cOR | aOR1 | aOR2 |
|---|---|---|---|
| Women | 1.04 (0.74-1.48) | | |
| Age | 0.94 (0.83-1.06) | | |
| Polymedication | 1.38 (0.97-1.98) | 1.80 (1.12-2.88) | |
| Concomitant infection | 1.52 (0.94-2.45) | | |
| Any SPS | 2.86 (1.88-4.37) | | |
| Any systemic/general SPS | 1.71 (1.20-2.44) | | |
| Persistent fever | 3.03 (1.72-5.32) | 2.23 (1.18-4.19) | 2.23 (1.18-4.20) |
| General malaise | 3.32 (1.65-6.68) | | |
| Pressure ulcers | 4.06 (1.40-11.74) | | |
| Any respiratory SPS | 1.78 (1.26-2.53) | 1.44 (1.00-2.14) | |
| Dyspnea | 1.71 (1.18-2.47) | | |
| Thoracic pain | 3.47 (1.95-6.18) | 2.55 (1.33-4.90) | 2.45 (1.26-4.77) |
| Headache | 2.05 (1.06-4.00) | | |
| Paresthesia | 2.40 (1.08-5.34) | | |
| Persistent anosmia or dysgeusia | 0.38 (0.14-0.91) | 0.28 (0.10-0.74) | 0.28 (0.10-0.74) |
| Any mental health SPS | 1.64 (1.01-2.67) | | |
| Any dermatological SPS | 2.45 (1.46-4.11) | 1.75 (1.01-3.03) | 1.79 (1.03-3.11) |
| Pruritus | 3.34 (1.36-8.21) | | |
| Exanthema | 2.44 (1.05-5.67) | | |
| Eczema | 4.04 (1.28-12.70) | 4.02 (1.16-13.92) | 3.97 (1.14-13.80) |
| Any nephrological SPS | 2.33 (1.15-4.70) | | |
| Any endocrinological SPS | 4.04 (1.28-12.70) | | |
| Glycemia uncontrol | 5.34 (1.18-24.11) | | |
| Any digestive symptoms | 1.78 (1.16-2.73) | | |
| Abdominal pain | 2.30 (1.19-4.44) | | |
| Hypotension or syncope | 2.61 (1.11-6.15) | | |
| Arrythmia or palpitations | 3.79 (1.64-8.77) | 3.08 (1.21-7.79) | 3.09 (1.22-7.85) |
| Superinfection | 2.54 (1.47-4.38) | 1.90 (1.05-3.42) | 1.79 (1.00-3.37) |
| Pneumonia | 8.03 (1.47-44.21) | 7.65 (1.27-45.97) | 7.87 (1.30-47.52) |

*cOR, odds ratios of the association between each variable and the return to Emergency Services during the 6 months after discharge for COVID-19 hospitalization; aOR1, odds ratios of the multivariant logistic regression models adjusted for all the variables of each category (sociodemographic, SPS, etc.); aOR2, odds ratios of the multivariant logistic regression models adjusted for all the considered variables of the database.*

**Table S4. Factors associated with hospital readmission during the 6 months after discharge for COVID-19 first hospitalization (n=35). Crude and adjusted logistic regression models.**

| Sequelae or persistent symptoms (SPS) | cOR | aOR |
|---|---|---|
| **Sociodemographic and clinical data on admission:** | | |
| Women | 1.55 (0.76-3.16) | |
| Age | 1.42 (1.09-1.85) | |
| Previous chronic kidney disease | 2.67 (0.90-7.86) | |
| Previous cardiovascular disease | 2.25 (1.06-4.76) | |
| Polymedication | 2.75 (1.31-5.79) | |
| Dependency | 5.46 (2.49-11.96) | |
| Not living in domicile | 5.69 (2.42-13.36) | |
| Normal TR at admission | 3.23 (1.35-7.70) | |
| Normal TR at discharge | 3.97 (1.31-12.04) | |
| Concomitant infection | 3.31 (1.36-8.06) | 7.68 (1.52-38.8) |
| CURB-65 | 2.04 (1.26-3.28) | |
| **Treatment during hospitalization:** | | |
| Hydroxychloroquine | 0.27 (0.10-0.71) | |
| Lopinavir/ritonavir | 2.13 (1.00-4.52) | |
| Tocilizumab | 2.84 (1.04-7.79) | 3.68 (1.18-11.49) |
| **SPS:** | | |
| Any systemic/general SPS | 3.23 (1.57-6.64) | |
| Persistent fever | 11.23 (4.33-29.14) | 8.31 (2.31-29.89) |
| Musculoskeletal pain | 2.52 (0.99-6.41) | |
| General malaise | 2.89 (0.86-9.54) | |
| Pressure ulcers | 10.84 (2.32-50.70) | |
| Any respiratory SPS | 3.22 (1.56-6.66) | |
| Dyspnea | 2.80 (1.33-5.90) | |
| Persistent cough | 2.30 (0.91-5.80) | |
| Headache | 5.15 (1.58-16-74) | |
| Disorientation or confusion | 10.84 (2.32-50.70) | |
| Any mental health SPS | 2.94 (1.14-7.56) | |
| Sleep disturbances | 3.98 (1.13-14.00) | |
| Any hematological SPS | 3.98 (1.13-14.00) | |
| Thrombotic phenomena | 8.10 (1.93-34.01) | |
| Any nephrological SPS | 14.81 (4.53-48.49) | 6.49 (1.50-28.14) |
| Renal insufficiency *de novo* | 11.86 (1.91-73.68) | |
| Any urological SPS | 7.88 (1.52-40.68) | |
| Digestive symptoms | 3.05 (1.29-7.20) | |
| Diarrhea | 4.19 (1.46-12.00) | |
| Constipation | 23.81 (2.40-235.82) | |
| Vomit | 15.39 (1.36-174.46) | |
| Hypotension or syncope | 3.89 (0.93-16.32) | |
| Superinfection | 8.10 (3.18-20.63) | 3.14 (1.05-9.40) |
| Urinary tract infection | 4.69 (1.07-20.55) | |
| Pneumonia | 11.86 (1.91-73.68) | 11.81 (1.40-99.39) |

*TR, thoracic radiography; cOR, odds ratios of the association between each variable and the readmission to hospitalization during the 6 months after discharge for COVID-19 first hospitalization; aOR, odds ratios of the multivariant logistic regression models adjusted for all the variables of each category, including sex and age for all the models.*

**Table S5. Factors associated with mortality during the 6 months after discharge for COVID-19hospitalization (n=8). Crude and adjusted logistic regression models.**

| Sequelae or persistent symptoms (SPS) | cOR | aOR |
|---|---|---|
| **Sociodemographic and clinical data on admission:** | | |
| Women | 1.95 (0.46-8.20) | |
| Age | 3.62 (1.72-7.59) | 3.57 (1.69-7.54) |
| Previous chronic kidney disease | 6.57 (1.54-28.11) | |
| Polymedication | 4.99 (1.00-24.90) | |
| Dependency | 9.74 (2.30-41.31) | |
| Living in residential centers | 5.24 (1.23-22.32) | |
| Not candidate to CPR | 16.4 (1.90-141.86) | |
| **Treatment during hospitalization:** | | |
| Hydroxychloroquine | 0.15 (0.04-0.64) | |
| Lopinavir/ritonavir | 0.08 (0.01-0.63) | |
| Azithromycin | 0.25 (0.06-1.03) | |
| **SPS:** | | |
| Persistent fever | 4.54 (0.89-23.02) | |
| Pressure ulcers | 21.6 (3.95-118.01) | 9.27 (1.49-57.65) |
| Any neurological SPS | 3.87 (0.96-15.64) | 4.82 (1.13-20.60) |
| Headache | 6.24 (1.22-31.91) | 14.32 (2.17-94.59) |
| Disorientation or confusion | 25.7 (5.7-115.84) | 8.58 (1.77-41.69) |
| Sleep disturbances | 6.77 (1.32-34.72) | |
| Any hematological SPS | 3.17 (0.38-26.51) | |
| Thrombotic phenomena | 11.10 (2.13-57.99) | 20.96 (2.98-147.54) |
| Any nephrological SPS | 13.75 (3.15-59.97) | 6.02 (1.25-28.95) |
| Any urological SPS | 7.89 (1.53-40.61) | 6.09 (1.10-33.61) |
| Otoacoustic symptoms | 12.38 (1.38-111.28) | 10.90 (1.00-118.47) |
| Constipation | 21.58 (3.95-118.00) | 15.32 (2.25-104.29) |
| Hypotension or syncope | 12.19 (2.32-63.98) | 8.52 (1.40-51.73) |
| Any superinfection | 7.29 (1.70-31.25) | |
| Pneumonia | 22.40 (2.31-217.32) | 28.55 (2.30-354.48) |

*cOR, odds ratios of the association between each variable and the readmission to hospitalization during the 6 months after discharge for COVID-19 first hospitalization; aOR, odds ratios of the multivariant logistic regression models adjusted for sex and age.*
